# Evaluation of vascular endothelial growth factor levels in tears and serum among diabetic patients

**DOI:** 10.1371/journal.pone.0221481

**Published:** 2019-08-22

**Authors:** Wen Jeat Ang, Embong Zunaina, Abdul Jalil Norfadzillah, Raja Omar Raja-Norliza, Muhammed Julieana, Siti Azrin Ab-Hamid, Mohamed Mahaneem

**Affiliations:** 1 Department of Ophthalmology, School of Medical Sciences, Health Campus, Universiti Sains Malaysia, Kubang Kerian, Kelantan, Malaysia; 2 Department of Ophthalmology, Melaka General Hospital, Jalan Mufti Haji Khalil, Melaka, Malaysia; 3 Unit Biostatistics and Research Methodology, School of Medical Sciences, Health Campus, Universiti Sains Malaysia, Kubang Kerian, Kelantan, Malaysia; 4 Department of Physiology, School of Medical Sciences, Health Campus, Universiti Sains Malaysia, Kubang Kerian, Kelantan, Malaysia; University of Florida, UNITED STATES

## Abstract

**Objective:**

Detection of vascular endothelial growth factor (VEGF) levels in ocular tissue may perhaps provide insight into the role of VEGF in the pathogenesis and progression of diabetic retinopathy (DR). The aim of this study was to evaluate the levels of VEGF in tears and serum amongst type 2 diabetes mellitus (DM) patients.

**Methods:**

A comparative cross-sectional study was conducted between August 2016 and May 2018 involving type 2 DM patients with no DR, non-proliferative DR (NPDR), and proliferative DR (PDR). Tear samples were collected using no.41 Whatman filter paper (Schirmer strips) and 5 mL blood samples were drawn by venous puncture. VEGF levels in tears and serum were measured by enzyme-linked immunosorbent assay.

**Results:**

A total of 88 type 2 DM patients (no DR: 30 patients, NPDR: 28 patients, PDR: 30 patients) were included in the study. Mean tear VEGF levels were significantly higher in the NPDR and PDR groups (114.4 SD 52.5 pg/mL and 150.8 SD 49.7 pg/mL, respectively) compared to the no DR group (40.4 SD 26.5 pg/mL, *p* < 0.001). There was no significant difference in the mean serum VEGF levels between the three groups. There was a fair correlation between serum and tear VEGF levels (*p* = 0.015, *r* = 0.263).

**Conclusion:**

VEGF levels in tears were significantly higher amongst diabetic patients with DR compared to those without DR and were significantly associated with the severity of DR. There was a fair correlation between serum and tear VEGF levels. Detection of VEGF in tears is a good non-invasive predictor test for the severity of DR. A large cohort study is needed for further evaluation.

## Introduction

Diabetic retinopathy (DR) is the leading cause of blindness in the working-age population and has a considerable economic burden on society, especially on healthcare systems [1,2]. In Malaysia, the 2015 National Health and Morbidity Survey (NHMS) estimated that 3.5 million (17.5%) Malaysians were diabetic and projected that number to increase to 21.6% by the year 2020 [3].

Microvasculopathy and inflammation are the main pathways involved in the pathogenesis of DR [4]. Studies have shown that the breakdown of the blood-retinal barrier is due to high levels of inflammatory mediators and cytokines [5,6–8]. Vascular endothelial growth factor (VEGF) is involved in the angiogenesis process of DR. VEGF has been associated with many other neovascular retinopathies, such as ischemic retinal vein occlusion and retinopathy of prematurity [9]. Levels of VEGF in the vitreous and aqueous humours are found to be higher during active proliferation of DR [9,10]. VEGF levels are reduced during the quiescent phase of proliferative diabetic retinopathy (PDR) and diminish after successful laser therapy [10].

The quantification of VEGF levels in the aqueous and vitreous humours in DR is reflecting the pathophysiological process due to closer proximity to the retina [10]. However, the measurement of aqueous and vitreous VEGF levels is rather invasive. It may cause complications, such as endophthalmitis or retinal detachment, and it is only ethically possible if performed during surgical intervention, such as cataract or vitreoretinal surgery. Quantification of VEGF levels is also done through blood sampling. Several studies found an inconsistent result of serum VEGF levels in DR patients [11–13]. Serum VEGF levels may be affected by systemic illnesses, such as hypertension [14] and dyslipidaemia, which are common comorbidities in diabetics [15], and also by smoking [16].

In recent years, tear analysis has become an increasing interest in ophthalmology. Detection of VEGF levels in tears is less invasive, safe and an acceptable method of screening for DR [17]. VEGF is found throughout the ocular epithelial structures, including in the cornea, ciliary body, lens and retinal pigment epithelium [18,19]. Moreover, the cornea epithelium has regenerative capability and is constantly shed with tears. In addition, tear fluid secreted by the lacrimal glands may also contain VEGF. Furthermore, VEGF which is found in the blood can permeate through the conjunctival vessels [20]. Detection of VEGF levels in tears may represent the pathophysiological changes of DR. Like serum, the level of VEGF in tears may differ according to the severity of DR, from non-proliferative diabetic retinopathy (NPDR) to PDR. The aim of this study was to evaluate and compare the levels of VEGF in serum and tears among type 2 diabetes mellitus (DM) patients.

## Methods

### Participants and selection criteria

A comparative cross-sectional study was conducted in the eye clinic of a tertiary centre from August 2016 to May 2018. The sample size was calculated using G Power 3.1.9. Type 2 DM patients aged 30 years and above were recruited using a systematic random sampling method. Exclusion criteria were patients: (a) with a history of ocular surface disease; (b) receiving systemic or topical steroid therapy within three months prior to recruitment; (c) receiving intravitreal therapy; (d) receiving laser photocoagulation therapy; (e) with a history of ocular trauma; (f) recovering from post-intraocular surgery (cataract, vitreous or retinal detachment) within six months prior to recruitment; and (g) with a history of any ocular inflammatory disease, such as uveitis. DR patients who presented with diffuse diabetic macular oedema and any patients who were being treated with anti-VEGF therapy were also excluded. Patients with systemic conditions that upregulate VEGF, such as bronchial asthma, chronic obstructive pulmonary disease, immunological disorder, haematological disease, malignancies or any condition associated with hypoxia or inflammation were excluded.

### Data collection

Demographic data (age, race and gender), acknowledgment of previous ocular surgery or treatment, systemic comorbidities (hypertension and dyslipidaemia) and smoking status (active, non-smoker and ex-smoker) was obtained through history-taking and medical records. Those who fulfilled the selection criteria were explained the nature of study and written consent was obtained.

All patients underwent a comprehensive ophthalmologic examination that included best-corrected visual acuity, slit-lamp examination, dilated fundus examination and applanation tonometry. Based on the International Clinical DR Disease Severity Scale (ICDRS) [21], patients were divided into three groups: no DR, NPDR and PDR. For DM patients with NPDR and PDR, only one eye of the worst severity was selected. In patients with PDR in one eye, tears were only collected from the PDR eye. DM patients with bilateral no DR were considered as a no DR group. Blood and tear samples were collected and measured for VEGF levels.

### Tear and blood sample collection

After a detailed explanation of the procedure to the patients, venepuncture was conducted and 5 mL of blood was collected. The blood samples were sent to a physiology laboratory. The blood samples were centrifugated at 1,000 rpm for 10 minutes to obtain the serum, which was then stored at −80°C prior to future analysis for VEGF.

Tears were collected using No.41 Whatman filter paper (Schirmer strips). Patients were asked to sit in an upright position with their backs well supported. A topical anaesthetic eye drop (Alcain-Alcon, Belgium) was instilled into the patient’s eye before the procedure. After 5 minutes, a Schirmer strip was inserted at the outer third of the lower eyelid. The patient was then asked to keep the eye gently closed until the tear wet the paper up to 15 mm. Then, forceps were used to remove the paper. The tear sample was kept in a clear 0.5 mL micro plastic test tube and sent immediately to a central research laboratory for storage. All samples were then kept at −80°C during the recruitment process.

Protein extraction from the Schirmer strips was performed by elution with triple-distilled water [22]. Each strip was cut into 3–5 mm pieces and placed in the same micro plastic test tubes. Then, 500 μL of ultrapure distilled water was added to this tube, which was centrifuged for 30 minutes at 10,000 rpm. The supernatant was transferred to another polypropylene tube and stored at −20°C until the following day, when the samples were thawed and centrifuged for 5 minutes at 14,000 rpm. The samples from the micro plastic test tubes were processed directly for analysis.

### VEGF quantification

A human VEGF immunoassay kit by Quantikine (R&D Systems, Minneapolis, MN, USA) was used for the determination of VEGF levels in serum and tears. The quantitative sandwich enzyme immunoassay technique was used according to the manufacturer’s instructions. For the initial step, 100 μL of RDW1 assay solution was placed onto the microplate. Then, 100 μL of the sample (serum and tear fluid) was added to the microplate and incubated for 2 hours at room temperature. Next, 200 μL of conjugate was added after three washings, followed by re-incubation at room temperature. Subsequently, it was subjected to three more washings before being centrifuged at 25 rpm, together with 200 μL of substrate, and incubated. The last solution that was added was 50 μL of stop solution. Finally, the plates were read using a microplate reader at 450–570 nm wavelength.

### Ethics approval

The study followed the tenets of the declaration of Helsinki and was approved by the local Human Research Ethics Committee (Universiti Sains Malaysia [USM]/Jawatankuasa Etika Penyelidikan Manusia [JEPeM]; Registration number: 16060224). Written and informed consent of participants was obtained for each patient prior to the study.

### Statistical analysis

The statistical analysis was carried out using Statistical Package for Social Sciences (SPSS) Version 24. A Chi-square test was used to compare gender and systemic illnesses (such as hypertension and dyslipidaemia) between the severities of DR. A Fisher’s exact test was used to compare race and smoking status between severities of DR. A one-way ANOVA test was used to compare mean age and duration of DM. An ANCOVA test was used to compare VEGF levels in tears and serum between severities of DR among type 2 DM patients, adjusted for age, duration of DM, smoking status, and systemic illness (hypertension and dyslipidaemia). For the comparison of the tears, a Bonferroni post-hoc test was used to compare VEGF among the three groups of DR patients. Simple linear regression and multiple linear regression were used to identify the associated factors for tear and serum VEGF. A Pearson correlation was used for the correlation of serum and tear VEGF. A *p*-value of < 0.05 was considered significant.

## Results

### Demographic data

A total of 88 type 2 DM patients (30 patients with NPDR, 28 patients with PDR, 30 patients with no DR) were recruited in this study. There were equal percentages of both males and females among the type 2 DM patients (n = 44, 50% for each group). Racial distribution showed that 84 patients (95.5%) were Malay and 4 patients (4.5%) were Chinese. The mean age of the enrolled patients was 58.5 SD 10.2 years and ranged from 52.3 to 64.6 years. There was significant difference in the mean age between the three groups (p = 0.021), where the PDR group had the youngest mean age (52.3 SD 9.6 years).

The duration of DM was longer in the PDR group compared to the no DR and NPDR groups, and there was significant difference among the three groups (p = 0.043). Hypertension (75.0%) and dyslipidaemia (70.5%) were the commonest systemic illnesses among DM patients. However, there was no significant difference among the three groups. The demographic data and systemic profiles among the three groups is shown in Table 1.

**Table 1:** Demographic characteristics and systemic profile among type 2 diabetic patients.

Variables	Total	No DR	NPDR	PDR	p-value
	(n = 88)	(n = 30)	(n = 28)	(n = 30)	
**Age** (years)					
Mean (SD)	58.5 (10.2)	64.6 (8.8)	58.5 (8.2)	52.3 (9.6)	0.021^a^
**Gender** (n, %)					
*Male*	44 (50.0)	17 (56.7)	11 (39.3)	16 (53.3)	0.377^b^
*Female*	44 (50.0)	13 (43.3)	17 (60.7)	14 (46.7)	
**Race** (n, %)					
*Malay*	84 (95.5)	28 (93.3)	27 (96.4)	29 (96.7)	0.950^c^
*Chinese*	4 (4.5)	2 (6.7)	1 (3.6)	1 (3.3)	
**Duration of DM** (years)					
Mean (SD)	9.2 (3.2)	7.7 (2.6)	9.9 (2.5)	10.1 (3.9)	0.043^a^
**Smoking Status** (n, %)					
*Non-smoker*	37 (42.0)	10 (33.3)	15 (53.6)	12 (40.0)	
*Active smoker*	37 (42.0)	14 (46.7)	11 (39.3)	12 (40.0)	0.462^c^
*Ex-Smoker*	14 (15.9)	6 (20.0)	2 (7.1)	6 (20.0)	
**Co-morbidity** (n, %)					
*Hypertension*	66 (75.0)	22 (73.3)	22 (78.6)	22 (73.3)	0.907^b^
*Dyslipidaemia*	62 (70.5)	21 (70.0)	19 (67.9)	22 (73.3)	0.956^b^

^a^ANOVA^,^

^b^Chi square test

^c^Fisher exact test, *p <* 0.05, significant

Abbreviation: DM: diabetes mellitus, DR: diabetic retinopathy, NPDR: non-proliferative diabetic retinopathy, PDR: proliferative diabetic retinopathy

### VEGF Levels in serum and tears

After adjusting for age, smoking status, duration of DM and systemic illness (hypertension and dyslipidaemia), the PDR group showed highest mean level of VEGF in serum among type 2 DM patients (342.4 SD 41.5 pg/mL) compared to NPDR (288.7 SD 34.8 pg/mL) and no DR (305.4 SD 43.0 pg/mL). However, there was no significant difference of mean serum VEGF levels between the three groups (*p =* 0.587) (Table 2).

**Table 2:** Comparison of mean VEGF level in serum and tears between the groups.

VEGF (pg/mL)	No DR	NPDR	PDR	F statistics	p-value
	(n = 30)	(n = 28)	(n = 30)	(df)	
**Serum VEGF**					
Mean (SD)	305.4 (43.0)	288.7 (34.8)	342.4 (41.5)	0.54 (2, 77)	0.587
**Tear VEGF**					
Mean (SD)	41.2 (11.3)	114.9 (8.6)	149.5 (10.4)	18.75 (2, 80)	<0.001

ANCOVA test applied after adjustment for age, smoking status, duration of DM, hypertension and dyslipidemia, *p* < 0.05, significant

Abbreviation: DR: diabetic retinopathy, NPDR: non-proliferative diabetic retinopathy, PDR: proliferative diabetic retinopathy, VEGF: vascular endothelial growth factor

Among type 2 DM patients, the NPDR and PDR groups showed a higher mean level of VEGF in tears (114.9 SD 8.6 pg/mL and 149.5 SD 10.4 pg/mL) compared to the no DR group (41.2 SD 11.3 pg/mL). There was a significant difference of mean tear VEGF levels between the three groups (*p <* 0.001) after adjusting for age, smoking status, duration of DM and systemic illness (hypertension and dyslipidaemia) (Table 2).

After the Bonferroni post-hoc analysis, there was a significant mean difference of tear VEGF levels between the no DR and NPDR groups (*p* < 0.001) and between the no DR and PDR groups (*p* < 0.001) (Table 3).

**Table 3:** Post Hoc comparison of mean tears VEGF after adjustment.

Variables	Group	Mean Difference (95%, CI)	p-value
	No DR–NPDR	68.9 (33.4, 104.6)	<0.001
Tear VEGF	No DR–PDR	99.8 (56.9, 142.7)	<0.001
	NPDR–PDR	30.8 (-0.9, 62.5)	0.060

Bonferroni Post-Hoc comparison test, *p* < 0.05, significant

Abbreviation: DR: diabetic retinopathy, NPDR: non-proliferative

The associated factors of serum and tear VEGF levels are summarised in Table 4. Age, duration of DM, severity of DR and VEGF levels were the significant variables among the associated factors with *p-*value < 0.25 based on simple linear regression. The four significant variables (*p* < 0.25) among the associated factors were included in the multiple linear regression. Smoking status was included, as it was of clinical importance. The results show that the tear VEGF level was a significant associated factor for serum VEGF (*p* = 0.015), whereas for the tear VEGF level, the severity of DR was a significant associated factor (*p* < 0.001).

**Table 4:** Associated factors of serum and tears VEGF.

Variables	Regression coefficient (b)	t-stats	p-value	Regression coefficient (b)	t-stats	p-value
	Simple Linear Regression*			Multiple Linear Regression**		
**Serum VEGF**						
Age	-3.10	-1.68	0.097	-0.09	-0.80	0.428
Gender	-9.89	-0.26	0.797			
Race	186.31	2.11	0.380			
Smoking status	-11.64	-0.63	0.533	-0.07	-0.66	0.514
Duration of DM	7.65	1.29	0.202	0.08	0.70	0.487
Hypertension	-38.08	-0.87	0.386			
Dyslipidaemia	31.58	0.75	0.454			
Severity of DR	54.16	2.43	0.017	0.14	0.91	0.364
Tears VEGF	0.71	2.49	0.015	0.71	2.49	0.015
**Tear VEGF**						
Age	-2.46	-3.94	<0.001	-0.55	-0.72	0.476
Gender	8.71	0.64	0.526			
Race	-18.31	-0.56	0.579			
Smoking status	0.47	0.07	0.944	1.63	0.34	0.737
Duration of DM	5.11	2.45	0.016	1.51	0.69	0.498
Hypertension	-13.14	-0.84	0.407			
Dyslipidaemia	-10.33	-0.69	0.493			
Severity of DR	55.23	9.54	<0.001	49.67	5.48	< 0.001
Serum VEGF	0.10	2.49	0.015	0.03	0.93	0.358

*Simple Linear Regression test, *p <* 0.25, significant

**Multiple Linear Regression test, p < 0.05, significant

Forward, Backward, Stepwise and Enter method were applied

Abbreviation: DM: diabetes mellitus, DR: diabetic retinopathy, VEGF: vascular endothelial growth factor

### Correlation of VEGF levels in serum and tears

**Fig 1:**
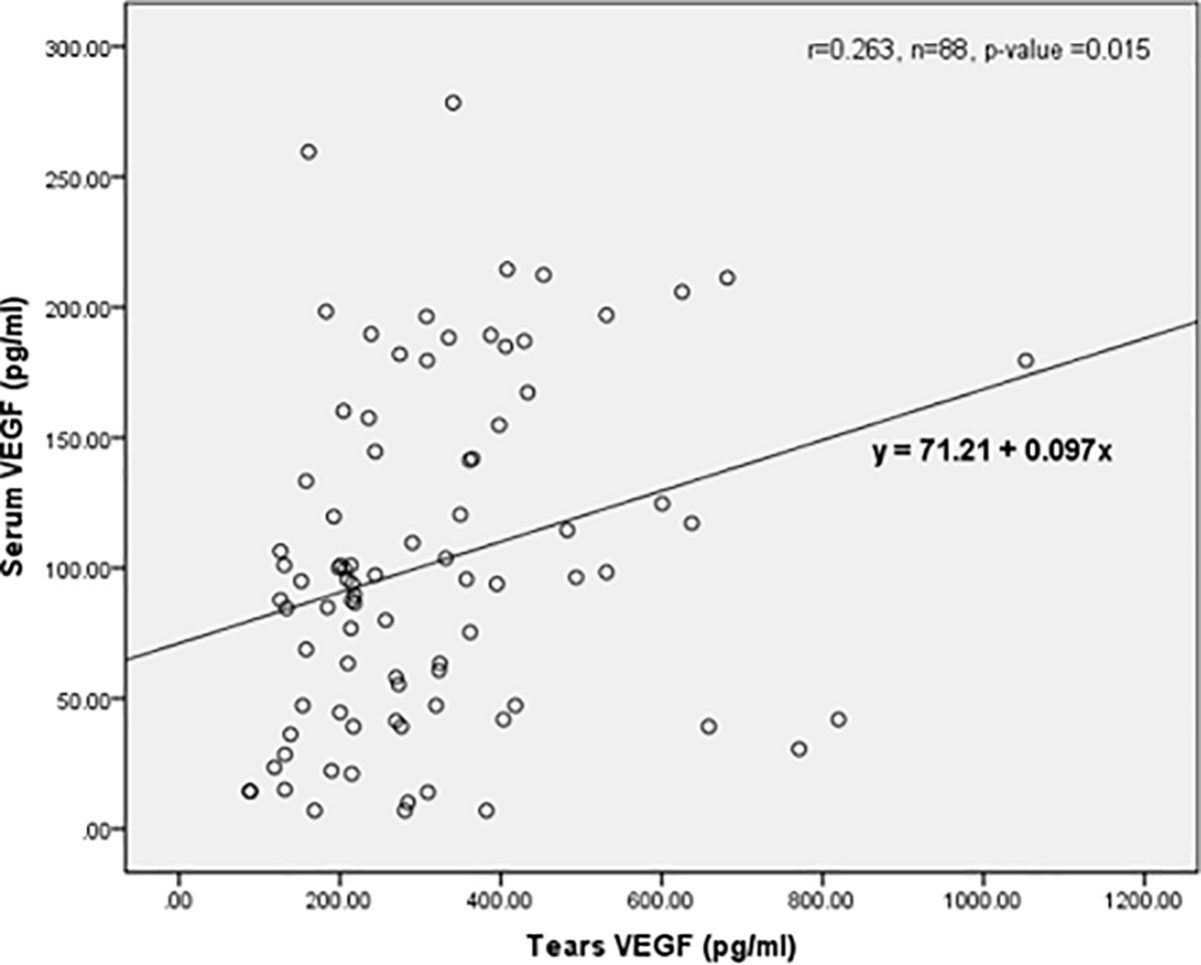
Correlation between serum and tears of VEGF level.

Fig 1 shows the correlation between serum and tear VEGF levels. There was a fair correlation between serum and tear VEGF levels (*p* = 0.015, *r* = 0.263).

## Discussion

In our study, we evaluate VEGF levels in serum and tears. In this study, a Schirmer strip was used for tear collection. According to Posa et al. [23], both a Schirmer strip and a capillary tube are highly suitable as gentle and non-invasive methods of sampling tear fluid from living subjects for efficient subsequent protein analysis. Furthermore, a Schirmer strip is easier to handle for the researcher and more pleasant to the patients.

We found that the mean serum VEGF of the no DR group was lower compared to the NPDR and PDR groups. Although the mean value of serum VEGF in the NPDR and PDR groups was higher than the mean value of VEGF in the no DR group, it was not statistically significant (*p* = 0.587). This is consistent with several studies done previously [11–13].

Serum levels of VEGF can be affected by the presence of other factors and systemic illness, which is common in diabetics. Some studies have suggested that hypertension [14], dyslipidaemia [15] and smoking [16] can influence serum VEGF levels. We also found a significant difference in the baseline characteristics, age (*p* = 0.021) and duration of DM (*p* = 0.043), of the three different groups (no DR, NPDR and PDR). The mean age of the patients was 58.5 SD 10.2 years and ranged from 52.3 to 64.6 years, which suggests that most of the type 2 DM patients are from the middle age groups, consistent with the common age of onset of type 2 DM. We found that the youngest age was observed in the PDR group. This group of patients may tend to present early when they are symptomatic due to complications of PDR, such as vitreous haemorrhage.

There is a strong relationship between duration of DM and stages of DR [24,25]. Although there are no pre-existing studies to link duration of DM and levels of VEGF, clinical data has shown that the longer the duration of diabetes, the more severe the stages of DR [26]. This is probably due to the longer duration of diabetic insult governed by various cytokines, such as interleukins and VEGF [27–29]. Therefore, adjustments to the possible confounding factors were made. After adjusting for age, smoking status, duration of DM and systemic illness (hypertension and dyslipidaemia), we found that there was no significance difference (*p* = 0.587) in serum VEGF levels. We speculate that the measurement of serum VEGF levels is non-specific and has limited use as biomarker for the prediction of DR. In addition, results of our study agree with other studies on different serum biomarkers of inflammation and haemostasis in association with DR after controlling for established risk factors [28–30]. Serum VEGF might not reflect the changes in the retina due to the relatively small size of affected tissue, and the amount of VEGF released in the large circulating blood volume [31].

On the other hand, we found that the mean tear VEGF levels were significantly higher (*p* < 0.001) in the PDR group and NPDR group compared with the no DR group after adjusting for age, smoking status, duration of DM and systemic illness (hypertension and dyslipidaemia). Interestingly, studies have demonstrated the presence of VEGF-like protein on the corneal epithelium [18,32]. Diabetic patients who are at a higher risk of developing diabetic keratopathy can easily shed corneal epithelium-containing VEGF proteins into the tear film, resulting in higher levels of VEGF [33,34]. Data on the role of the lacrimal gland in the secretion of VEGF via the tear aqueous layer is still lacking; nonetheless, studies on animals have shown us that significant VEGF is detected in the lacrimal glands of rat models [35]. This data could also explain the elevated tear film VEGF levels. A study done on central retinal vein occlusion patients found that eyes with retinal vein occlusion demonstrated higher levels of VEGF in tears compared to normal fellow eyes [36]. These findings support that there is an association between a hypoxic retina and the level of VEGF in tears [36]. Likewise, in our study, mean tear VEGF levels were higher in the patients with DR.

We are unable to compare our findings of tear VEGF levels, as thus far there have been no studies done to evaluate VEGF levels in tears among type 2 DM patients. Nevertheless, our results are comparable with a study done by Ermakova et al. on type 1 DM patients, which shows a relationship between the tear VEGF level and the degree of various manifestations of DR [37]. Tear VEGF levels may also be more representative of the ocular level of VEGF, as they are in closer proximity and share a similar circulation [38]. However, tear VEGF levels may be exposed to atmospheric changes. The influence of these changes on tear VEGF levels is not well studied yet. Possibly, to improve the reliability of our results of tear VEGF levels, an assessment of dry eye syndrome could be included in future studies. Dry eye syndrome is not only relatively common among DM patients, but it could influence the level of VEGF in the tears by reducing tear volume [39].

Our findings on tear VEGF is also in line with previous reports on other ocular fluid, such as aqueous and vitreous VEGF, although aqueous and vitreous samples are shown to yield a higher mean VEGF level in PDR patients [40–45]. This could be due to the proximity of the anterior chamber and vitreous cavity to the retina. Furthermore, aqueous and vitreous VEGF measurement is likely to eliminate any confounding factors on the ocular surface. The limitation of this study is we did not compare tears to aqueous humour and vitreous humour samples. This comparison might not only enhance our understanding of VEGF and its interactions in the eye but also solidify the association of VEGF in ocular fluids and DR. In view of that, we suggest a concurrent evaluation of VEGF levels in the aqueous humour or vitreous humour and ocular tear film VEGF levels in future studies. Aqueous and vitreous samples can probably be collected prior to cataract surgery and intravitreal anti-VEGF therapy, respectively. Another major drawback of our study is we did not include haemoglobin A1c (HbA1c) levels. Correlating HbA1c with serum and tear VEGF levels will give us a better overview of the control of DM and its relationship with systemic and ocular VEGF levels. We strongly recommend this in future studies.

In the extension of our study, we also found that VEGF levels in serum and tears were associated with age, duration of DM and severity of DR. The VEGF level in serum was associated with the VEGF level in tears, and vice versa. Further, multiple linear regression analysis revealed that tear VEGF was the independent predictor of the VEGF level in serum of this cohort (*p* = 0.015). The analysis also showed that severity of DR was the only independent predictor of VEGF levels in tears of our patients. This indicates an association among severity of DR and tear VEGF in type 2 DM patients.

In our study, we also observed a fair correlation between serum and tear VEGF levels (*p* = 0.015 and *r* = 0.263). This suggests that an interaction may exist between serum and tear VEGF. However, the relationship between serum VEGF and tear VEGF remains to be investigated. Studies have shown that VEGF is known to increase microvascular permeability by raising calcium influx into the endothelial cells which form the vessel walls [46,47]. We postulate that VEGF in the blood circulation can get into the tear fluid through increased permeability of the conjunctival vessels. Despite this, we did not see a significant difference in mean serum VEGF levels amongst our patients. This could be explained by a study done by Burgos et al. [43]; in their study, they believe that ocular VEGF originates inside the eye. This could be due to the relatively small structure, such as the affected retina tissues, in comparison with the amount of VEGF released in the large circulating blood volume [31]. This correlation remains debatable. A larger sample size in future studies might be helpful.

## Conclusion

Among type 2 DM patients, VEGF levels in tears were higher amongst patients with DR compared to those without DR. Tear VEGF levels were also significantly related to the severity of DR. There was a fair correlation between serum and tear VEGF levels. In the future, tear VEGF levels might be used as a non-invasive tool to expedite screening programs and to predict the severity of DR in patients with diabetes. A large cohort study is needed for further evaluation.

## Abbreviations

DM: Diabetes mellitus
DR: Diabetic retinopathy
HbA1c: Hemoglobin A1c
ICDRS: International Clinical Diabetic Retinopathy Disease Severity Scale
JEPeM: Jawatankuasa Etika Penyelidikan Manusia
NHMS: National Health and Morbidity Survey
NPDR: Non-proliferative diabetic retinopathy
PDR: Proliferative diabetic retinopathy
SD: Standard deviation
SPSS: Statistical Package for Social Sciences
USM: Universiti Sains Malaysia
VEGF: Vascular endothelial growth factor
